# Jean Alfred Fournier

**Published:** 1915-02

**Authors:** 


**Jean Alfred Fournier** died in Paris, December 24, aged 82. He was a professor of the medical faculty of Paris and a well known dermatologist and syphilographer.

